# Combining Anti-ERBB3 Antibodies Specific for Domain I and Domain III Enhances the Anti-Tumor Activity over the Individual Monoclonal Antibodies

**DOI:** 10.1371/journal.pone.0112376

**Published:** 2014-11-11

**Authors:** Jimson W. D’Souza, Smitha Reddy, Lisa E. Goldsmith, Irina Shchaveleva, James D. Marks, Samuel Litwin, Matthew K. Robinson

**Affiliations:** 1 Molecular Therapeutics Program, Fox Chase Cancer Center, Philadelphia, PA, United States of America; 2 Biostatistics and Bioinformatics Facility, Fox Chase Cancer Center, Philadelphia, PA, United States of America; 3 Department of Anesthesia and Pharmaceutical Chemistry, University of California San Francisco, San Francisco, California, United States of America; Bauer Research Foundation, United States of America

## Abstract

**Background:**

Inappropriate signaling through the epidermal growth factor receptor family (EGFR1/ERBB1, ERBB2/HER2, ERBB3/HER3, and ERBB4/HER4) of receptor tyrosine kinases leads to unregulated activation of multiple downstream signaling pathways that are linked to cancer formation and progression. In particular, ERBB3 plays a critical role in linking ERBB signaling to the phosphoinositide 3-kinase and Akt signaling pathway and increased levels of ERBB3-dependent signaling is also increasingly recognized as a mechanism for acquired resistance to ERBB-targeted therapies.

**Methods:**

We had previously reported the isolation of a panel of anti-ERBB3 single-chain Fv antibodies through use of phage-display technology. In the current study scFv specific for domain I (F4) and domain III (A5) were converted into human IgG1 formats and analyzed for efficacy.

**Results:**

Treatment of cells with an oligoclonal mixture of the A5/F4 IgGs appeared more effective at blocking both ligand-induced and ligand-independent signaling through ERBB3 than either single IgG alone. This correlated with improved ability to inhibit the cell growth both as a single agent and in combination with other ERBB-targeted therapies. Treatment of NCI-N87 tumor xenografts with the A5/F4 oligoclonal led to a statistically significant decrease in tumor growth rate that was further enhanced in combination with trastuzumab.

**Conclusion:**

These results suggest that an oligoclonal antibody mixture may be a more effective approach to downregulate ERBB3-dependent signaling.

## Introduction

The ERBB family of receptor tyrosine kinases (RTKs) is comprised of the epidermal growth factor receptor (EGFR/ERBB1), ERBB2/HER2, ERRB3/HER3, and ERBB4/HER4. In normal epithelial-derived tissues, signaling through this family of RTKS is regulated through ligand-driven homo- and heterodimerization. Each family member exhibits unique features making them essential for defined cellular processes. Thus, appropriate expression patterns of receptors and their ligands are required for normal tissue homeostasis. Unregulated and/or inappropriate signaling through EGFR and ERBB2, as a consequence of protein overexpression or mutation, is linked to both formation and progression of a variety of epithelial-derived tumors [1]. As such, EGFR and ERBB2 have been the focus of extensive drug development efforts and are the targets of both small-molecule tyrosine kinase inhibitors (TKIs) and antibody-based therapies that are FDA-approved for treating a variety of indications, including breast (BrCa), lung, colorectal, head and neck, and gastric cancers (GCa) [2].

Phosphoinositide-3-kinase (PI3K) and Akt signaling play a central role in coordinating the regulation of a variety of cancer relevant processes, including cellular metabolism and proliferation. Because of this, aberrant signaling through the PI3K/Akt pathway is implicated in the formation and progression of many cancers, including ERBB-driven cancers [3]. Downregulation of PI3K/Akt signaling induced by EGFR and ERBB2 targeted therapies correlates with the anti-proliferative effect of these agents [4]. The ERBB family members contain unique structural features that result in non-overlapping functions [1]. For example, ERBB3, in contrast to other family members, contains six consensus phosphotyrosine binding sites within its C-terminal tail for the p85 regulatory subunit of PI3K. Transphosphorylation of these sites promotes recruitment of PI3K and subsequent activation of Akt [5], [6]. Therefore, ERBB3 represents a major intersection point between ERBB signaling and the PI3K/Akt pathway. Despite its direct link to the PI3K/Akt pathway ERBB3, unlike EGFR and ERBB2, cannot induce cellular transformation on its own [7]. However, the importance of ERBB3 activity in promoting ERBB-driven cancers is suggested by its ability to cooperate with ERBB2 to enhance cellular transformation [8] and the tumor regression associated with short hairpin RNA-based (shRNA-based) knockdown of ERBB3 in mouse models of ERBB2-positive breast cancer (BrCa) [9]. Likewise, oncogenic *ERBB3* mutations have been identified in a number of diseases, including ERBB2-positive BrCa and GCa, but the growth promoting activity of those mutations are dependent upon ERBB2 activity [10].

ERBB3 was historically ignored as a drug target due, in part, to its enzymatically inactive kinase domain [11]. This feature precluded its targeting with small molecule tyrosine kinase inhibitors (TKIs), such as erlotinib and lapatinib that are used to block EGFR and ERBB2 signaling. ERBB3 levels are also not amplified in comparison to those seen with either EGFR or ERBB2. However, as its integral role in linking ERBB signaling to the PI3K/Akt pathway has become more defined, antibody-based approaches are being developed to inhibit ERBB3 signaling. Both traditional monoclonal antibodies [12], [13], [14], [15], [16] and next generation molecules like bispecific antibodies [17], [18], [19] and alternative scaffolds [20] are at various stages of preclinical and clinical development and represent promising approaches to inhibiting ERBB3 activity. However, the development of resistance to anti-EGFR (e.g. cetuximab) and anti-ERBB2 (e.g. trastuzumab) antibodies in the clinical setting suggests that more effective strategies are necessary to fully block signaling through this family of receptors. One approach being investigated for EGFR and ERBB2 is the use of oligoclonal mixtures of antibodies (≥2 non-competitive mAbs) as a treatment option. Mixtures of either anti-EGFR or anti-ERBB2 mAbs have been shown to be more effective than single mAbs in preclinical studies [21], [22], [23]. More recently, the combination of anti-ERBB2 antibodies, trastuzumab and pertuzumab, was shown to improve clinical efficacy [24], [25]. To our knowledge the use of oligoclonal mixtures of mAbs to enhance efficacy associated with targeting ERBB3 has not been previously investigated. In this study we demonstrate that an oligoclonal mixture of anti-ERBB3 mAbs is more effective than individual component mAbs at inhibiting tumor cell growth and suggests an approach for more effective downregulation of ERBB3-dependent signaling.

## Materials and Methods

### Cell culture

BT-474 (ATCC # HTB-20), SK-BR-3 (ATCC# HTB-30), NCI-N87 (ATCC# CRL-5822), ACHN (ATCC# CRL-1611) and HEK293 (ATCC# CRL-1573) were purchased from the American Tissue Culture Collection. SK-BR-3 and HEK293 cells were cultured in DMEM/HEPES, BT-474 cells were cultured in DMEM/F12 and NCI-N87 and ACHN cells were cultured in RPMI-1640 media. Cells were cultured at 37°C and 5% CO_2_ in media supplemented with 10% heat-inactivated fetal bovine serum (FBS), 2 mM L-glutamine and antibiotics–100 I.U./ml penicillin and 100 µg/ml streptomycin.

### Epitope mapping

Cloning and expression of intact ERBB3 ECD (dI-IV, aa20–639) and dIII-IV (aa310–616, a kind gift of Ralf Landgraf) are described elsewhere [26], [27]. DNA fragments corresponding to aa20–207 (dI) and aa20–329 (dI-II) were amplified by PCR on AscI/NotI fragments, sub-cloned into pSEC-Tag2/HygroA (Invitrogen) and stably expressed as C-terminally tagged 6X–His fusion proteins in HEK293 cells. A DNA fragment corresponding to aa311–477 (dIII) was amplified on a BglII/XbaI fragment, subcloned into pMTBiPV5HISa and stably expressed as a C-terminally tagged 6X–His fusion protein in S2 cells. In all cases proteins were purified from culture supernatant by IMAC over a HisTrap column (GE Healthcare, cat #17-5319-01). Proteins were stored frozen at −80°C in 20% glycerol.

A5 and F4 scFv were conjugated to agarose resin using commercially available AminoLink reagents (Pierce, cat #20381) to facilitate domain level mapping of the antibody epitopes by immunoprecipitation (IP). Briefly, 6.4 mg of A5 and F4 scFv were buffer exchanged into 2 mL of pH7.2 coupling buffer and used to resuspend pelleted AminoLink resin slurry (1 mL of packed resin/antibody). Single-chain antibodies were then incubated with AminoLink resin overnight at 4°C. Resins were pelleted, washed with quenching buffer, and remaining sites inactivated by incubation in quenching buffer at room temp for 30 minutes. Amount of A5 and F4 coupled to beads was determined from the concentrations of A5 and F4 in pre-coupling and post-coupling fractions as determined with a colorimetric assay (BioRad, cat #500–0001). Resin containing approximately 80 µg of bound scFv was incubated for one hour at room temperature with 5 µg of an appropriate ERBB3 ECD fragment (20–50 fold excess of scFv) in a final volume of 40 µL. Resin was pelleted and 30 µL of the supernatants, representing unbound fraction, was collected. The immobilized antibody/ECD complexes were washed with 3×1 mL aliquots of PBS and resuspended in 30 µL PBS. Equal fractions of bound and free scFv were analyzed by SDS-PAGE. As appropriate, bands were quantified by densitometry using ImageJ software (NIH) and normalized to controls.

Reactions containing IgG and dI were additionally electrophoresed in a 12.5% acrylamide gel under native conditions using the PhastGel System (GE Healthcare, cat #17-0623-01) and stained to detect proteins and protein complexes. F4 and A5 IgG (30–40 µg) were incubated in a 4 µL binding reaction with dI at 1.5x and 3x Molar ratios of IgG:dI by varying the amount of dI. Binding was allowed to occur for 30 minutes at room temperature. Incubation of dI with PBS (2 µL) served as an additional negative control.

### *In vitro* efficacy

Cells were plated in 96-well plates (Corning, cat #3595) in triplicate at densities (2000–5000 cells/well) that resulted in vehicle-treated wells failing to reach confluence at the end of the experiment. Cells, growing in media supplemented with 1% FBS, were treated as defined in individual figures and cell viability, normalized to vehicle-treated controls, was measured using CellTiter blue (Promega, cat #G8081) and manufacturer’s recommended protocol.

### Inhibition of signal transduction

To assess inhibition of basal signaling, cells were seeded at a density of 500,000 cells/well in 48-well plates and allowed to adhere overnight. Cells were serum starved in 0.5% serum containing media for 12–16 hours and treated with 1.6 nM trastuzumab, 100 nM cetuximab, 1 µM A5, 1 µM F4, PBS or combination of two or three antibodies for prescribed time.

To assess the ability of antibodies to block ligand-induced signaling, cells were seeded at a density of 250–500,000 cells/well in 48-well plates and allowed to adhere overnight. After overnight serum starvation in 0.1–0.5% serum containing media, cells were pretreated with appropriate antibodies or PBS for 30 minutes and stimulated with 20 nM HRG-beta (R&D systems #377-HB-050) for indicated times.

In both situations cells were washed twice with cold phosphate buffered saline and lysed in 1X lysis buffer (cell signaling #9803) supplemented with protease (Thermo scientific #1861279) and phosphatase (Roche #04906845001) inhibitor cocktails. Lysates were cleared from cell debris by centrifugation at 16,000×g in cold for 30 minutes and protein concentrations measured as described previously.

### Western blotting

Rabbit phospho-ERBB3 (Y1289) (#4791), ERBB3 (#12708P), phospho-ERBB2 (Y1221/1222) (#2243), phospho-Akt (Ser473) (#4060) and mouse ERBB2 (#2248) and Akt (pan) (#2920) antibodies were from Cell Signaling Technology. Mouse anti-beta actin monoclonal antibody was from Sigma-Aldrich. Horseradish peroxidase-conjugated goat anti-rabbit and donkey anti-mouse secondary antibodies were from Jackson ImmunoResearch. Donkey anti-mouse-IRDye® 680CW and goat anti-rabbit-IRDye® 800CW were from Licor imaging systems.

Equal amounts of protein for each lysate were electrophoretically separated on 4–12% Bis-Tris gels (Life Technologies) and transferred onto polyvinylidene membrane. Membranes were blocked with either 1X Odyssey blocking buffer or 5% BSA in Tris-Buffered Saline with 0.1% Tween-20 for 2 hours at room temperature. Appropriate primary antibodies were diluted in the blocking buffer for probing the membranes. For Odyssey detection, donkey anti-mouse-IRDye® 680CW and goat anti-rabbit-IRDye® 800CW polyclonal secondary antibodies were used and the blots were visualized on Licor OdYssey infrared imaging system. For conventional western blot, the membranes were probed with horseradish peroxidase-conjugated goat anti-rabbit and donkey anti-mouse secondary antibodies and developed with SuperSignal West Pico chemiluminescent substrate (Thermo scientific #34080). Band pixel densities were quantified using ImageJ software (NIH). Densitometric values were background subtracted, normalized to respective beta-actin signal and signal relative to PBS-treated samples was plotted using GraphPad PRISM software.

### Xenograft efficacy studies

Male NCR nude mice harboring NCI-N87 tumor xenografts (120–150 mm^3^) were randomly assigned into cohorts and treated with appropriate drug or vehicle for four weeks. Trastuzumab was administered once every week at 4 mg/kg body weight, equivalent to the clinical dose level. The anti-ERBB3 mAbs were each injected at 20 mg/kg body weight twice a week intraperitoneally, consistent with literature for other ERBB3 mAbs [13]. Tumor volume measurements were performed twice a week using the formula: (longer measure)^2^×shorter measure)/2. These studies were carried out under a protocol approved by the Fox Chase Cancer Center IACUC committee.

For western blot studies tumor-bearing mice were treated with the appropriate drug or vehicle and euthanized 4 hours post-treatment. Tumors were immediately harvested, sliced into sections, flash frozen in liquid nitrogen and stored at −80°C. Tumor slices were homogenized on ice in 1∶20 w/v T-Per tissue protein extraction reagent (Pierce #78510) supplemented with protease (Thermo scientific #1861279) and phosphatase (Roche #04906845001) inhibitor cocktail. Tissue debris was separated by centrifugation at 16,000×g in cold for 45 minutes and supernatants were cleared through 0.45 µm syringe filter unit. Protein concentrations were measured as previously described and equal amounts of protein were analyzed by western blot.

### Statistical methods

The combination index of A5/F4 with either trastuzumab or erlotinib against each of 4 cell lines was determined using the equation CI = D_1/_ID_X,1_+D_2/_ID_X,2_. In each case 100 bootstrap estimates of the combination index (CI) were each compared to 1.0 and the number equaling or exceeding 1.0 was tallied. The p-value is this tally divided by the number of boots (100). CI<1 indicates synergism if significant. Tumor growth was analyzed using the Wilcoxon Rank Sum test. Briefly, each animal’s data was divided by 100, log transformed and the slope of the best fitting exponential determined. Each animal was then represented by this slope or growth rate. The rates were submitted to a Wilcoxon test and significance determined.

## Results

### Construction and Testing of antibodies specific for domain I and domain III of ERBB3

**Figure 1:**
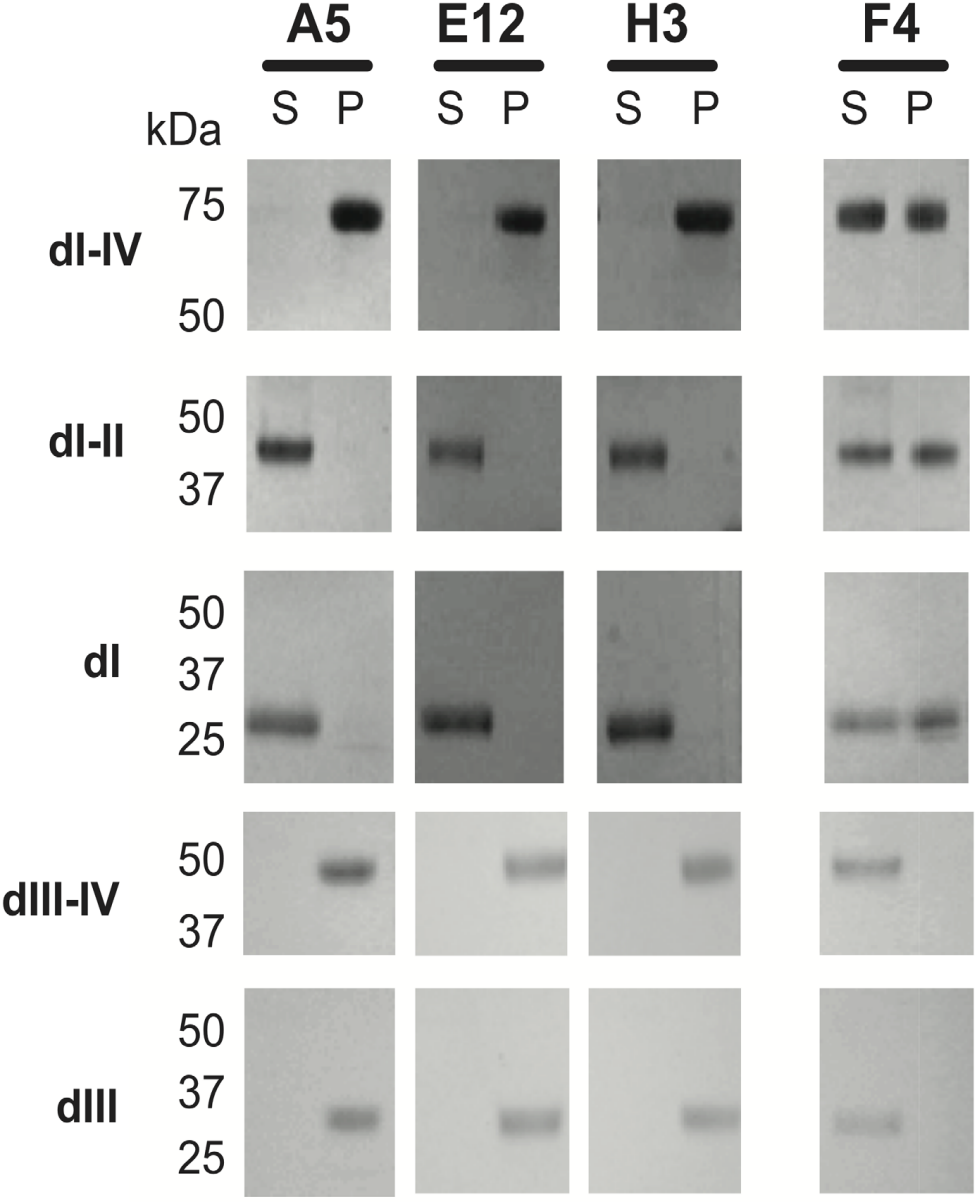
Ability of single-chain Fv molecules to inhibit cell viability correlates with epitope. A) Intact (dI-IV) and sub-domains (dI, dI-II, dIII, dIII-IV) of ERBB3 extracellular domain were expressed and subjected to immunoprecipitations with immobilized scFv under conditions of antibody excess. S  =  supernatant (unbound fraction), P  =  pellet (bound fraction).

We previously identified a panel of scFv specific for the extracellular domain (ECD) of ERBB3 by panning a naïve human phage display library [26]. The ECD of ERBB3 shares a similar architectural structure with other family members being composed of four domains (henceforth denoted as dI, dII, dIII, and dIV, in N- to C-terminal orientation) linked by flexible regions. The dI and dIII domains are compact leucine-rich domains with an overall globular structure that together comprise the ligand-binding pocket of the receptor. Domains II and IV are cysteine-rich, rod-like structures. Members of the panel of anti-ERBB3 scFv (A5, E12, F4, and H3) were immobilized on agarose beads and analyzed for their ability to immunoprecipitate (IP) both intact, as well as subdomains of the ERBB3 ECD. As depicted in Figure 1A, all four scFv immunoprecipitated the intact ERBB3 ECD (dI-IV). Using truncated versions of the ERBB3 ECD we demonstrated that dIII was necessary and sufficient for A5, H3, and E12 scFv to IP ERBB3 ECD. In IP-based competition studies, soluble A5, H3, and E12 scFv competed the binding of intact ERBB3 (dI-IV) to their respective scFv immobilized on agarose beads (Figure S1). In addition, under the conditions used in the IP studies, these scFv exhibited varying degrees of reciprocal competition, suggesting that they bind either partially overlapping or closely positioned epitopes on dIII (Figure S1). The F4 scFv represented the sole member of a second class of anti-ERBB3 scFv. The F4 antibody binds to an epitope located on dI; dI was necessary and sufficient for immunoprecipitation of ERBB3 ECD constructs (Figure 1, Figure S2). Consistent with binding a spatially distinct epitope F4 failed to compete A5 binding to intact ERBB3 ECD (Figure S1).

**Figure 2:**
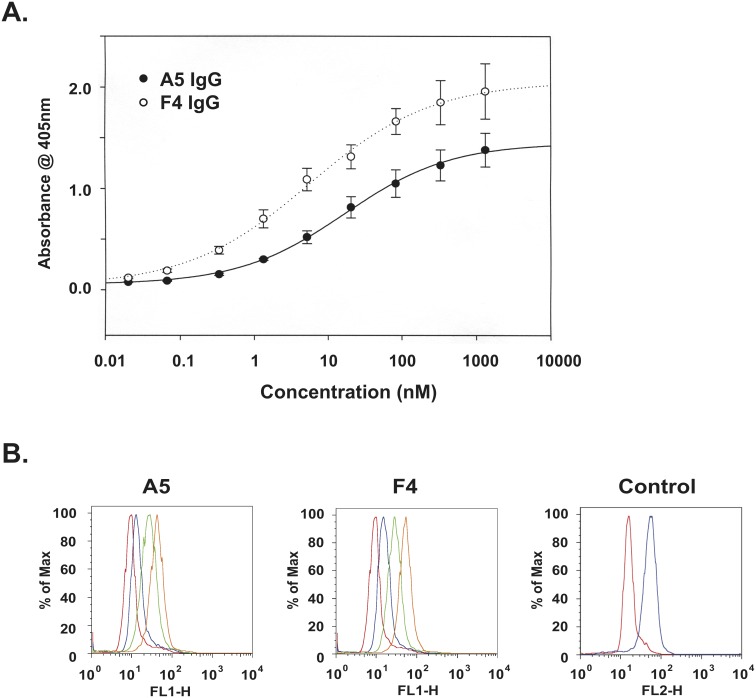
Binding activity of A5 and F4 anti-ERBB3 IgGs. A) Equilibrium binding of A5 and F4 IgGs to purified ERBB3 extracellular domains was quantified in an ELISA format by incubating 4 fold-serial dilutions of IgG (1333–0.02 nM) and 0 nM control with immobilized ERBB3, and bound antibody was detected with HRP-conjugated anti-human Fc secondary antibody. Binding to ERBB2 was used to control for specificity. Values plotted represent the means ± standard deviation of a representative experiment B) Increasing amounts (0, 0.5, 2, 5 µg) of A5 and F4 IgG were incubated with BT-474 cells (250,000 cells/assay) and binding was detected by flow cytometry with a FITC-conjugated anti-human Fc secondary antibody. Cells were incubated with 2 µg of PE-conjugated anti-ERBB3 antibody (SGP1-PE) as a positive control. Equivalent amounts of mouse IgG1-PE (R&D Systems, cat #IC002P) served as a negative control.

Because of the potential therapeutic relevance associated with targeting the ligand-binding domains of ERBB3 we constructed human IgG1 versions of the F4 and A5 scFv as representative dI and dIII-binders (Figures S3–S5). In ELISA-based equilibrium binding studies the A5 and F4 IgGs exhibited apparent affinities of 11.1±1.6 nM and 4.8±2.2 nM, respectively (Figure 2A). These values represent 10–40 fold increases in apparent affinity over the 167 nM and 211 nM intrinsic affinities of A5 scFv [19] and F4 IgG (Figure S6) determined by surface Plasmon resonance (SPR). These increases in apparent affinity, likely due to avid binding, are similar to those seen with other IgG constructed from low affinity scFv antibodies [28]. Flow cytometric analysis with BT-474 cells suggests the epitopes for both A5 and F4 are exposed on the cell surface. Equivalent shifts in the Mean Fluorescent Index (Figure 2B) were obtained with both increasing concentrations of A5 and F4. Those shifts were similar to those seen with a commercially available anti-ERBB3 antibody.

### An oligoclonal mix of anti-ERBB3 antibodies blocks ligand-induced ERBB3 signaling

**Figure 3:**
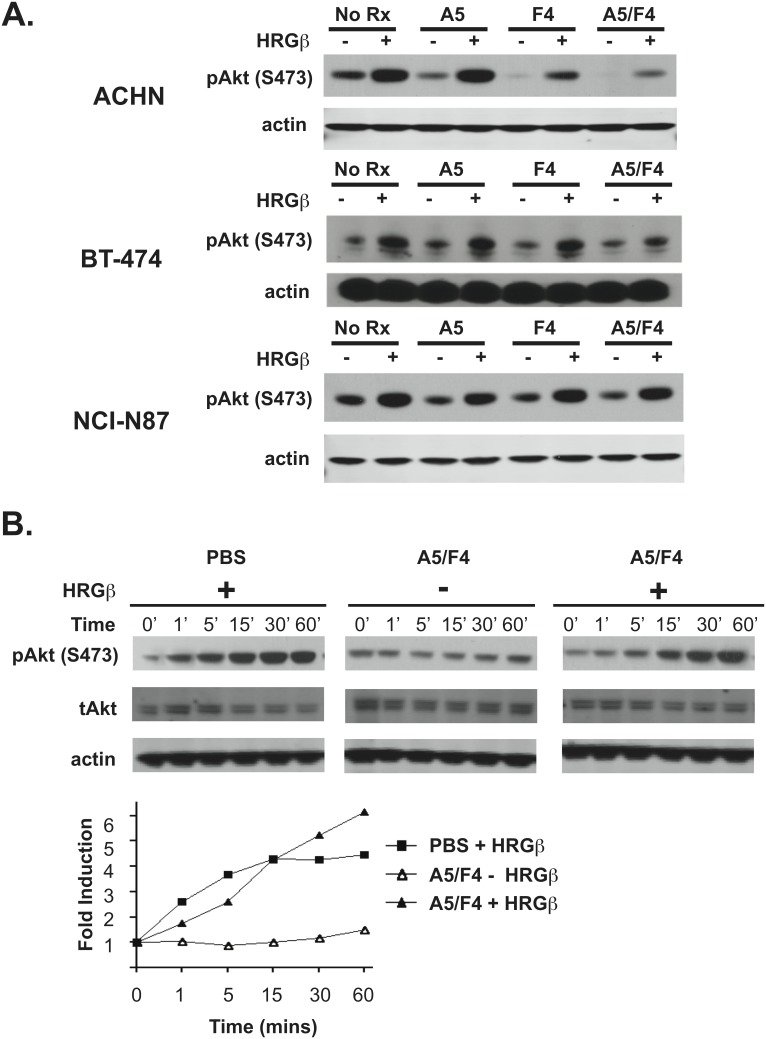
Anti-ERBB3 mAbs inhibit ligand-mediated ErbB3 signaling. A) Serum-starved BT-474, NCI-N87 and ACHN cells were treated with appropriate antibodies or PBS for 30 minutes followed by HRGβ stimulation for 10 minutes. B) NCI-N87 cells were treated with either the A5/F4 oligoclonal or PBS and stimulated with HRGβ, as appropriate, over a 60 minute time course. Cell lysates were analyzed by western blot and probed with anti-pAKT(S473) as a marker for ligand-induced signaling. Membranes were probed with anti-beta actin mAb that served as loading control.

The specificity of F4 and A5 for dI and dIII, respectively, led us to investigate their ability to block ligand-induced ERBB3 signaling in the heregulin-responsive BT-474, NCI-N87 and ACHN cell lines. BT-474 and NCI-N87 cells represent ERBB2-positive and ACHN cells represent EGFR-positive models. These cell lines exhibit a low level of pAKT(S473) under serum starvation conditions that is induced within 10 minutes of heregulin (HRGβ) stimulation (Figure 3A). Pretreatment of ACHN cells, and to a lesser degree BT-474 and NCI-N87 cells, with either A5 or F4 partially blocked this HRGβ-dependent increase in pAKT(S473) levels. Importantly, in both ERBB2 and EGFR-positive models the A5/F4 oligoclonal is capable of more effectively blocking ligand-dependent upregulation of pAKT(S473) levels than either A5 or F4 IgG alone. Together this data indicates that A5, F4, and to a larger extent the A5/F4 oligoclonal, are capable of blocking HRGβ-dependent signaling *in vitro*. However, HRGβ can overcome the A5/F4-dependent blockade of ERBB3 signaling in a time-dependent manner. This is exemplified in Figure 3B where initial HRGβ-induced levels of pAKT(S473) are reduced in A5/F4 treated NCI-N87 cells as compared to PBS controls. However, by 15–30 minutes, levels of pAKT(S473) in A5/F4 treated cells have reached those observed in non-treated cells by 1–5 minutes of ligand stimulation. This data suggests that affinity maturation of A5 and F4 may be necessary to optimize overall efficacy of the A5/F4 oligoclonal.

### The A5/F4 oligoclonal mix inhibits basal levels of ERBB3 signaling

**Figure 4:**
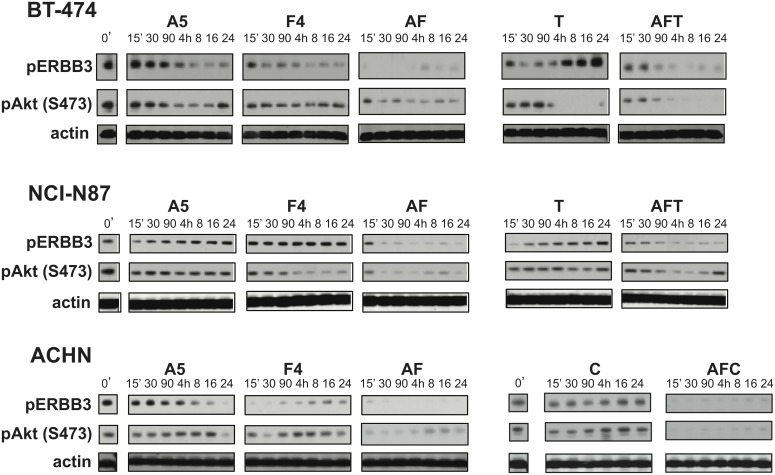
Effects of anti-ErbB3 mAbs on basal ErbB3 signaling. BT-474, NCI-N87 and ACHN cells growing in complete media were serum starved for 12–16 hours and treated with 1 µM A5, 1 µM F4, 1.6 nM trastuzumab (T), 100 nM cetuximab (C), PBS or combinations of A5/F4 (AF), A5/F4/trastuzumab (AFT), or A5/F4/Cetuximab (AFC) for a prescribed time. Cell lysates were analyzed by western blot with pERBB3(Y1289) and pAKT(S473) mAbs as markers of ERBB3 signaling, as well as anti-beta actin mAb as a loading control.

We further examined the mechanisms of action associated with A5, F4, and the A5/F4 oligoclonal by investigating their ability to block basal signaling through the ERBB3 receptor as measured by levels of pERBB3(Y1289) and pAKT(S473). Although the magnitude of effect associated with A5 and F4 IgGs varied between cell types when they were used as single agents the A5/F4 oligoclonal consistently demonstrated the greatest ability to block basal signaling (Figure 4). Treatment of BT-474, NCI-N87, and ACHN cells with the A5/F4 oligoclonal altered the basal levels of activated ERBB3 and AKT as assessed by levels of pERBB3(Y1289) and pAKT(S473) as compared to untreated controls.

In general F4 IgG appeared more proficient at downregulating initial signaling events as assessed by its ability to decrease levels of pERBB3(Y1289) and pAKT(S473). Treatment of BT-474 cells with either A5 or F4 resulted in a time-dependent decrease in the levels of pERBB3(Y1289), and to a lesser extent pAKT(S473). Similar trends were observed upon treatment of ACHN cells, with F4 having a more dramatic impact on pERBB3 levels than in either of the ERBB2-positive cell lines. Exposure of NCI-N87 cells to either A5 or F4 did not show significant effect on pERBB3(Y1289), however treatment with F4 did correspond to decreased pAKT(S473) levels in a time dependent manner. This suggests that phosphorylation of ERBB3 on one of its five other consensus p85 binding sites may be more critical for downstream activation of AKT in NCI-N87 cells. As seen with ligand-induced signaling, the A5/F4 oligoclonal exhibited a more pronounced effect on decreasing levels of both pERBB3(Y1289) and pAKT(S4573) as compared to either A5 or F4 in all cell lines examined.

Treatment of ERBB2-amplified BT-474 and NCI-N87 cell lines with trastuzumab led to a rapid loss of pERBB3(Y1289) levels in cells that rebounded to baseline within a few hours of treatment (Figure 4). This time-dependent reactivation of ERBB3 and somewhat paradoxical lack of a concomitant increase in pAKT(S473) levels we observed in Figure 4 is consistent with data reported by Garrett and colleagues where they demonstrated phosphorylation on T308 rather than S473 correlated with ERBB3 reactivation in BT-474 cells [29]. Interestingly, in contrast to BT-474 cells the level of pAKT(S473) in NCI-N87 cells was maintained upon trastuzumab treatment. However, in both cell lines the addition of the A5/F4 oligoclonal to trastuzumab reduced ERBB3 reactivation as denoted by the prolonged decrease in pERBB3 levels. In addition, the level of pAKT(S473) was reduced in both BT-474 and NCI-N87 cells as compared to treatment with trastuzumab alone. This is consistent with the idea that direct targeting of ERBB3 may improve overall response. Treatment of ACHN cells with cetuximab had a similar, albeit more modest, effect on ERBB3 and AKT as seen with trastuzumab treatment in BT-474 and NCI-N87 cells; treatment resulted in a short-term decrease in levels of pERBB3(Y1289) and pAKT(S473) that was lost over the course of the experiment. However, addition of the A5/F4 oligoclonal to cetuximab treatment enhanced the extent and time of pAKT inhibition, similar to what was seen with the A5/F4 plus trastuzumab in ERBB2-driven cancer cells.

### The A5/F4 oligoclonal inhibits cell growth and enhances activity of ERBB2 and EGFR-targeted therapies

**Figure 5:**
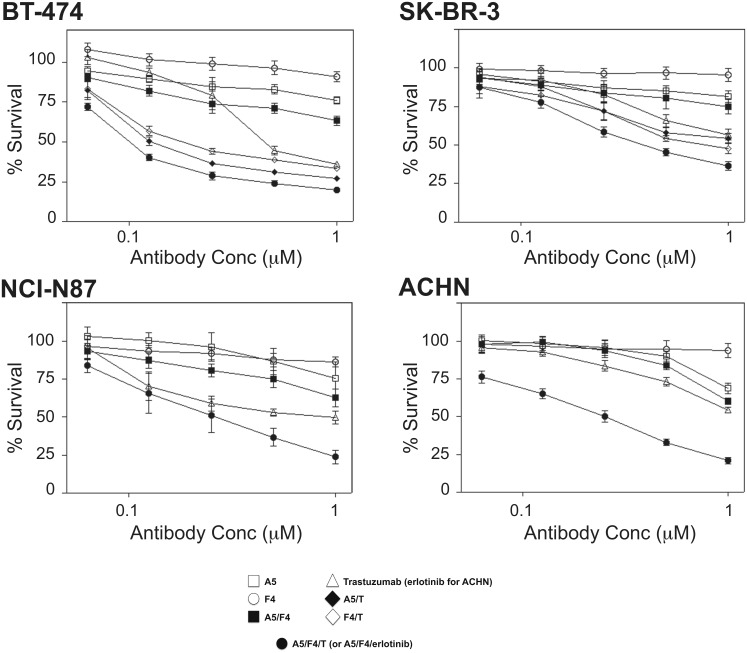
Combining A5 and F4 IgGs improves growth inhibition of ERBB3-positive cell lines and synergizes with ERBB-targeted agents. Individual, pairwise, and triple combinations of A5, F4 and either trastuzumab or erlotinib (as appropriate) were serially diluted (2-fold dilution scheme) and incubated with the ERBB2-positive cell lines, BT-474, SK-BR-3, NCI-N87, and EGFR-positive cell line ACHN, as noted. Concentration plotted on the x-axis refers to anti-ERBB3 IgG concentration. Trastuzumab and erlotinib are at 1∶200 molar ratios with anti-ERBB3 antibodies. The impact of treatment on cell viability was quantified with Cell-Titer Blue. Vehicle-treated controls for each cell line were set at 100%. Values plotted represent the means ± standard error of the means (SEM) for between 2–6 independent experiments, each carried out in triplicate.

We next investigated if the ability of the dI (F4) or dIII (A5) specific IgGs to block signaling correlated with the ability to inhibit growth of a panel of ERBB2-positive breast (BT-474 & SK-BR-3) and gastric (NCI-N87) cells as well as the EGFR-positive renal cancer cell line (ACHN). Although the absolute level of inhibition varied between cell lines, treatment with A5 IgG induced a modest, dose-dependent, level of growth inhibition (Figure 5).

Analogous to the improved signal inhibition, treatment of BT-474 (Figure 5A) and NCI-N87 (Figure 5C) cells with an equimolar mixture of A5/F4 improved cell growth inhibition over treatment with either A5 or F4 IgGs alone at all but the lowest concentrations tested. The oligoclonal exhibited, at best, a modestly enhanced activity against SK-BR-3 (Figure 5B) and ACHN (Figure 5D) cells when compared to A5 alone. Together this data suggests the A5/F4 oligoclonal, when used as a single agent to simultaneously target dI- and dIII, can be more effective at inhibiting the growth of cells dependent upon ERBB3 signaling than treatment with either antibody alone.

In agreement with the published literature, treatment of BT-474, SK-Br-3, and NCI-N87 cells with increasing concentrations of trastuzumab (0–5 nM) resulted in a dose-dependent decrease in cell viability (Figure 5). Blockade of ERBB3-dependent signaling in combination with ERBB2 blockade has been shown to improve cell growth inhibition [16]. As compared to either agent alone the combination of the A5/F4 oligoclonal plus trastuzumab at an empirically determined fixed ratio (200∶1) synergistically improves cell growth inhibition at the 50% effect level in all three cell lines tested (Figure 5). The combination indices (CI) associated with treating BT-474 (Figure S7A) and NCI-N87 (Figure S7C) with the A5/F4 oligoclonal plus trastuzumab were calculated to be 0.225 (p<0.01) and 0.128 (p<0.01), respectively. Although less sensitive to A5/F4 oligoclonal as a single agent, A5/F4 still synergized with trastuzumab (CI = 0.492, p<0.01; Figure S7B) to inhibit the growth of SK-Br-3 cells to similar levels as seen in BT-474 and NCI-N87 cells. Likewise, A5/F4 synergized with the EGFR-selective tyrosine kinase inhibitor erlotinib at the 50% effect level (CI = 0.403, p<0.01) to inhibit growth of the erlotinib-responsive ACHN cells (Figure 5D, Figure S7D).

Interestingly, despite having no activity as a monotherapy in the cell growth assay the addition of F4 to trastuzumab enhanced trastuzumab’s impact on cell growth. Treatment of BT-474 cells with increasing concentrations of a mixture of F4/trastuzumab at the same fixed ratio (200∶1) resulted in a similar, albeit consistently lower, level of enhanced growth inhibition as compared to the A5/trastuzumab mixture. Similar results were also seen with SK-Br-3 cells. However, the magnitude of the enhancement was greater in BT-474 cells than SK-Br-3, consistent with the sensitivity of the cells to A5 therapy. In both cell lines the A5/F4 oligoclonal was more effective at enhancing trastuzumab activity than either anti-ERBB3 IgG alone.

### The A5/F4 oligoclonal mixture of ERBB3 antibodies demonstrates *in vivo* activity alone and in combination with trastuzumab

**Figure 6:**
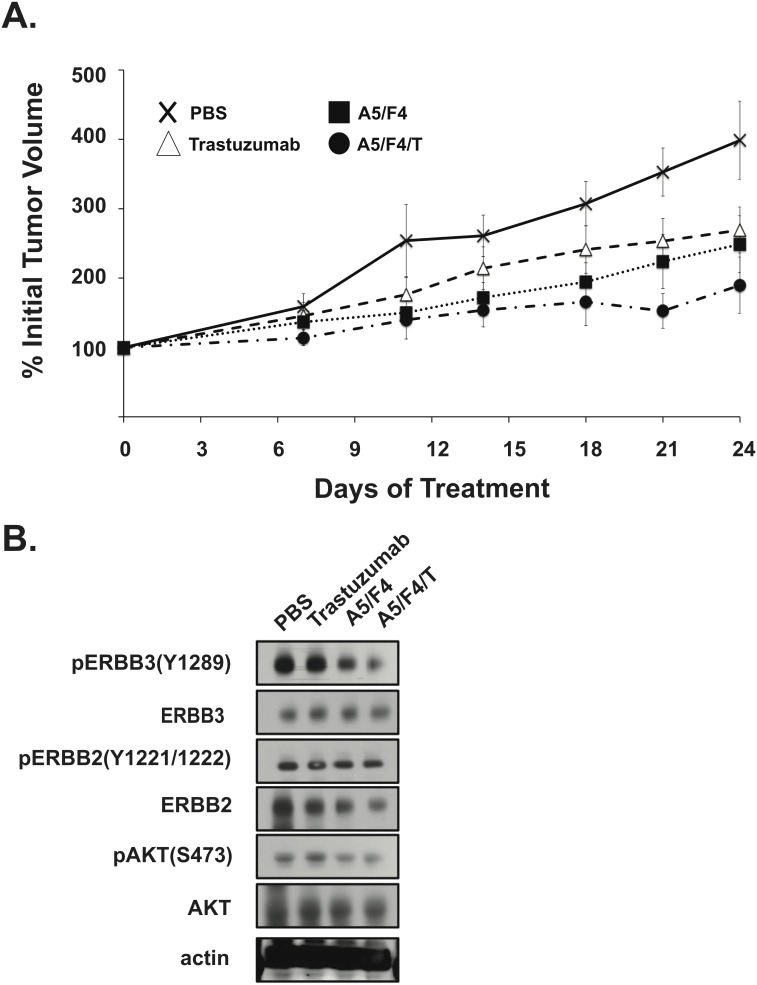
The A5/F4 oligoclonal exhibits in vivo efficacy. NCR nu/nu mice harboring 120–150 mm^3^ NCI-N87 subcutaneous tumor xenografts were randomly assigned into treatment groups (n≥6/group) and received i.p injections as described in Methods. Means +/−SEM of percentage tumor volume growth is shown for each measurement. Statistical analysis was performed with Wilcoxon Ranked Sum test.

The ability of the A5/F4 oligoclonal to both block signaling and inhibit tumor cell growth *in vitro* supported analysis of its *in vivo* efficacy. To this end we chose to examine the efficacy of the A5/F4 oligoclonal alone and in combination with trastuzumab using the NCI-N87 ERBB2+ gastric cancer xenograft model, as inhibiting ERBB3 in the setting of ERBB2+ gastric cancer represents a promising approach for the treatment of this disease. When administered at a dose and schedule equivalent to that used for preclinical testing of an anti-ERBB3 antibody that has progressed to clinical development [13], the A5/F4 oligoclonal (20 mg/Kg each, BIW) significantly reduced (p = 0.004) tumor growth rates as compared to vehicle treatment (Figure 6A). When administered at clinically relevant doses, trastuzumab (4 mg/Kg, q wk) also inhibited growth of the NCI-N87 tumors to a statistically significant level as compared to vehicle treatment (p = 0.02). At the dose levels and schedules used in these studies, targeting ERBB3 with the A5/F4 oligoclonal induced a similar level of growth inhibition as observed with trastuzumab (p = 0.35). Consistent with *in vitro* results demonstrating that addition of A5/F4 to trastuzumab is more effective at inhibiting tumor cell growth the A5/F4/trastuzumab combination also trended toward improved tumor growth control. At the dose levels and schedules employed, the addition of A5/F4 to trastuzumab improved tumor growth control (p = 0.08) when compared to trastuzumab alone. Because of the trend toward improved response we postulated that this enhancement would correspond with downregulation of biomarkers of ERBB3 signaling in the xenograft model. As seen in Figure 6B, Western blot analysis of tumor lysates prepared 4 hours after treatment suggests that the A5/F4 oligoclonal is capable of decreasing levels of both pERBB3 and pAKT as compared to vehicle treated controls. Under the same conditions trastuzumab treatment exhibited no major impact on pERBB3, pERBB2 and pAKT levels. However, treatment with the A5/F4 oligoclonal plus trastuzumab caused a further reduction in pERBB3 and pAKT levels over either treatment alone. The A5/F4/Trastuzumab (AFT) combination also appeared to reduce total levels of ERBB2 but not ERBB3. The combination of tumor growth reduction and pERBB3 levels observed in tumor lysates suggests that A5/F4 are capable of blocking ligand mediated ERBB3 signaling *in vivo*.

## Discussion

Our expanding appreciation of the mechanisms by which ERBB3 acts to transduce signaling when heterodimerized with other ERBB-family members and the role it plays in the development of drug resistance (reviewed in [30]) has led us, and others to develop antibody-based approaches to disrupt signaling through this receptor. Here we report for the first time the efficacy associated with targeting ERBB3 with an oligoclonal mixture of two IgGs specific for domains I and III. We have also demonstrated that this mixture is capable of enhancing the activity of ERBB2-targeted agents both *in vitro* and *in vivo*.

Crystal structure data of the extracellular domain of EGFR alone and in complex with its ligand EGF depict both locked (dimerization refractory) and extended (dimerization competent) conformations, respectively. These studies suggest a model where ligand-driven signaling through EGFR, and by extension ERBB3, occurs via binding of ligand to dI and dIII, which stabilizes the extended conformation and promotes receptor dimerization necessary for activation of downstream signaling pathways [31], [32]. The data suggests that in the setting of ERBB2 amplified cancers, overexpression of ERBB2 leads to constitutive ligand-independent heterodimerization with ERBB3 and activation of downstream signaling events that promote tumor progression [9]. In this setting dual blockade of ERBB2 with either a combination of trastuzumab plus pertuzumab or trastuzumab plus lapatinib is more effective at inhibiting signaling downstream of ERBB2. However, the importance of inhibiting ERBB3-driven signaling directly is exemplified in a study by Garrett et al demonstrating that dual blockade of ERBB2 signaling via co-treatment with both trastuzumab plus lapatinib fails to fully inhibit signaling through ERBB3 [33]. The ability of ERBB3 to signal through both ligand-dependent and ligand-independent mechanisms suggests development of agents capable of inhibiting both mechanisms may be necessary for optimal efficacy and broadest applicability.

Unlike the A5/F4 oligoclonal, which appears capable of inhibiting both ligand-independent and ligand-induced signaling, the anti-ERBB3 mAbs currently in clinical trial (MM-121 and AMG 888) block ligand-dependent signaling through the receptor [13], [34]. A recent preclinical report describes LJM716, an anti-ERBB3 mAb that binds a conformational epitope comprised of dII and dIV and is capable of inhibiting both ligand-independent and ligand-stimulated signaling via ERBB3 [35]. This mechanism of action is presumably similar to the anti-EGFR antibody matuzumab that is theorized to block signaling by preventing the receptor from adopting the extended conformation [36]. The model of ligand-binding inferred from the EGFR crystal structure, and consistent with the ability of F4 IgG to block ligand binding, suggests that antibodies directed against dI could have the potential to block ligand-dependent signaling through these receptors in a manner similar to that observed for antibodies that bind dIII. This may be particularly true for ERBB3 as biochemical data suggests that dI of ERBB3 plays a more prominent role in binding to HRGβ, an ERBB3 ligand, than dI of EGFR plays in binding its ligand epidermal growth factor (EGF) [27]. Landgraf and colleagues demonstrated that HRGβ binding to ERBB3, in contrast to EGF binding to EGFR, is retained under conditions of low pH (pH 5.5) that approximate the conditions experienced by the receptors during internalization. In fact, the interaction between dI and HRGβ is increased at pH 5.5 as compared to neutral pH. Consistent with this model, Lazrek et al recently described dI-binding anti-ERBB3 antibodies capable of blocking HRG-induced phosphorylation of Akt [16]. However, its ability to enhance the activity of ERBB2-targeted agents suggests its binding impacts the balance between activating and inhibitory mechanisms dictating ERBB3 activity. As described here the A5 and F4 IgG are capable of blocking phosphorylation of ERBB3 and AKT over the initial 24 hours of treatment but interestingly F4’s activity fails to translate into inhibition of cell growth when used as a single agent. The exact mechanism for this apparent discrepancy is not fully understood. One possible explanation is that F4’s epitope only provides for partial steric inhibition of HRGβ binding. This would allow HRGβ to outcompete F4 over the course of the cell growth assay, leading to sufficient ERBB3 activation to promote growth. However, F4’s ability to potentiate the activity of other ERBB-targeted agents necessitates that it partially inhibits ERBB3-dependent signaling over the course of cell growth assays. Efforts are underway to more fully map the epitopes of both A5 and F4 to provide a structural basis for the difference in activities seen between these two antibodies and among the dI- and dIII-binding antibodies described in the literature.

It is envisioned that approaches to down-regulate ERBB3 levels on the cell surface will block both ligand-dependent and ligand-independent signaling mechanisms and may prevent the resistance to ERBB2-targeted TKI treatments that are associated with increased levels of ERBB3 [37]. Single anti-ERBB3 mAbs are capable of causing ERBB3 internalization but as seen with MM-121 can have disparate effects on ERBB3 depending on the cell context. Treatment of ACHN renal carcinoma cells led to rapid drop in pERBB3 levels without an effect on total ERBB3 levels. In contrast, treatment of NCI-N87 gastric cancer cells resulted in loss of ERBB3 from the cell surface [13]. Combinations of antibodies are more effective at down-regulating other members of the ERBB family [21], [22], [23]. We hypothesize that oligoclonal mixtures of ERBB3-specific antibodies will be more effective than single mAbs at driving internalization and degradation of ERBB3, potentially limiting the cellular differences seen above. In addition, internalization and degradation of ERBB3 is hypothesized to block both ligand-dependent and ligand-independent signaling mechanism. Our results demonstrating more robust inhibition of cell growth by the A5/F4 oligoclonal, both alone and in combination with other ERBB-targeted agents, are consistent with the hypothesis that the oligoclonal is more effective at inhibiting ERBB3-dependent signaling. Although western blot data from tumor samples suggest that A5/F4 had no impact on total levels of ERBB3 in the xenografts this does not rule out increased internalization of the receptor. Data demonstrating the exact mechanism driving this response, and its applicability across cancer types, remains to be determined.

In comparison to MM-121 (∼0.8 nM, [13]) and AMG 888 (∼1 nM, [34]) the A5 and F4 IgGs bind to ERBB3 with low affinity. Despite the low affinities the A5/F4 oligoclonal was capable of inhibiting tumor xenograft growth both as a single-agent and in combination with trastuzumab. This was seen in spite of the fact that exogenous HRGβ can override the A5/F4-induced blockade of ERBB3-dependent signaling when analyzed *in vitro*. HRGβ is endogenously overexpressed in 30% of invasive breast cancers, and its overexpression correlates with increased levels of pERBB2 in 67% of the cancers even though ERBB2 is expressed at low levels [38]. HRGβ overexpression in a large percentage of invasive breast cancers, and its ability to reduce the *in vitro* efficacy of trastuzumab against BT-474 cells [39], emphasizes the need to effectively compete for ligand-dependent activation of ERBB3. Therefore, it is likely that further development of these mAbs will require affinity maturation but the optimal affinity for these antibodies is unclear. Preclinical evidence suggest that high affinity may not be appropriate in all situations as it can inhibit the IgG from penetrating into the tumor and potentially limit efficacy. Evaluation of a panel of anti-ERBB2 IgGs that bind the same epitope with varying affinities showed dramatic differences in tumor penetration [40]. The total amount of antibody delivered to the tumors was independent of affinity but higher affinity antibodies failed to penetrate long distances from the vasculature. Rather, higher affinity antibodies were more readily internalized and catabolized by tumor cells surrounding the vasculature, preventing their penetration. Clinical data generated with the anti-EGFR antibody nimotuzumab (2.1×10^−8^ M monovalent affinity [41]) suggests that moderate affinity can be sufficient for efficacy and may promote more selective tumor targeting and decreased systemic toxicities by requiring divalent binding of two EGFR molecules as part of its mechanism of action (reviewed in [42]). This requirement for divalent binding to improve targeting is analogous to the principle we successfully employed in developing a bispecific single chain Fv (bs-scFv) antibody to selectively target tumors that express both ERBB2 and ERBB3 [19]. It is important to note that the impact of affinity on targeting and efficacy of anti-EGFR and anti-ERBB2 antibodies has been described under conditions where the target antigens are expressed at 10–100x higher levels than those seen with ERBB3.

In summary, we have described the novel approach of inhibiting ERBB3 function through use of an oligoclonal mixture comprised of dI-binding (F4) and dIII-binding (A5) antibodies. This mixture exhibited both *in vitro* and *in vivo* efficacy. Our data suggests that an oligoclonal antibody approach to inhibiting ERBB3 signaling, analogous to that described for other ERBB family members, may be more efficacious than a traditional mAb-based approach. Future studies are focused on defining an optimal mixture of antibodies for enhancing ERBB3 downregulation across the broadest spectrum of diseases.

## Supporting Information

Figure S1 **Epitope binning of anti-ERBB3 scFv.** A5, E12, and H3 anti-ERBB3 scFv were immobilized on agarose beads and incubated with intact ERBB3 ECD (dI-IV) in the presence of excess free scFv (A5, E12, H3, or F4) to compete for binding as depicted. The impact of competing scFv on immunoprecipitation of dI-IV was evaluated by SDS-PAGE analysis of supernatant and pellet fractions. Numbers obtained by densitometric analysis of the supernatant and pellet fractions represent the percent of dI-IV that was immunoprecipitated by the immobilized ‘IP antibody’ in the presence of excess free ‘competing antibody.’
(TIF)

Figure S2 **F4 IgG can quantitatively bind dI.** F4 and A5 IgGs (30–40 µg) were incubated with dI (2.5–6 µg) at 1.5x and 3x Molar excess of antibody for 30 minutes at room temperature. Binding reactions were resolved on 12.5% native acrylamide gel. As compared to input dI (incubated with equal volume of PBS), incubating with increasing ratio of F4:dI shows dose dependent loss of free dI consistent with quantitative binding. A5 failed to bind under either condition tested.
(TIF)

Figure S3 **Amino acid sequence of the A5 and F4 variable domains.** Amino acid sequences of the variable heavy (Vh) and variable light (Vl) domains for the A5 and F4 IgGs are provided in single letter code. Complementarity Determining Regions (CDRs) are based upon definitions described by North et al [43].
(TIF)

Figure S4 **Purification of A5 and F4 IgGs.** SDS-PAGE analysis of A5 and F4 IgG purification by protein A chromatography. Arrowheads denote heavy (∼50 kDa) and light (∼25 kDa) chains of expressed IgGs. Media  =  conditioned media containing expressed IgG, Elution  =  IgG eluted from protein A column, F.T.  =  flow through fraction from protein A column, Mrkrs  =  molecular weight markers.
(TIF)

Figure S5 **Protein Thermal Shift assay of A5 and F4 IgG.** In thermal stability assays the A5 (top panel) and F4 (middle panel) scFv and IgGs were quantified using the Protein Thermal Shift assay (Life Technologies) and manufacturer’s recommended conditions and software. The A5 and F4 IgGs exhibited single transition points at 68.3±0.2°C and 69.2±0.3°C, respectively. This represented a stabilization over the A5 scFv (Tm = 63.3±0.1°C) and was equivalent to the F4 scFv that itself exhibited a Tm of 70±0.2°C. Trastuzumab and an scFv (4D5) engineered based on the trastuzumab amino acid sequence served as controls (bottom panel). Trastuzumab exhibited two major transition points at approximately 70°C and 83°C when analyzed by PTS that are consistent with the 68°C and 80°C melting point transitions obtained by differential scanning calorimetry [44]. Fluorescence and d(Fluorescence)/dT are plotted as a function of temperature.
(TIF)

Figure S6 **BIAcore analysis of the F4 IgG intrinsic binding affinity.** ERBB3 binding by F4 IgG was analyzed by surface Plasmon resonance using a capture-based strategy. F4 IgG (approx 150 RU) was captured on an anti-human Fc surface followed by ERBB3 dI-IV being flowed over the surface. Binding was analyzed at increasing concentrations of ERBB3 dI-IV, in duplicate, and data was fit to a 1∶1 binding model using BIAevaluation.
(TIF)

Figure S7 **Combination Indices of A5/F4 plus standard of care ERBB-targeted agents.** Survival fraction versus dose data for the several drug combinations were fitted using three parameter Hill equations: S(d) = A+(1–A)/[1+(d/d0)∧p], where d is the applied dose and A, d0 and p are parameters. The dose for the fitted equation S(d) = 0.5 was determined for each drug combination and identified as the ID50. Drugs that could not reach 50% kill according to the fitted equation were assigned an infinite ID50 for CI computation. ID50 dose levels, where possible, are indicated in the figure. The combination index was found from CI = d1/D50(1)+d2/D50(2) where LD50(1) or LD50(2) are the estimated ID50s of the single or part-combination of two or the three drugs used. d1 and d2 are the ID50 of the corresponding drugs or part combinations that result in 50% kill using the triple drug combination. In figure 7S–D ACHN cells were inhibited at the 50% level by both the A5/F4 combination alone or by Erlotinib alone. In this case the two ID50s were taken from the first and second plots of D. Synergistic dose, d, was determined from the third plot. In the other three cell lines either the A5/F4 or the Trastuzumab never reach 50% kill. In these one of the two fractions was considered zero by virtue of the assumption that its ID50 was infinite.
(TIF)

Materials and Methods S1 (DOC)
