# Supplementary figures and images for: Combining Anti-ERBB3 Antibodies Specific for Domain I and Domain III Enhances the Anti-Tumor Activity over the Individual Monoclonal Antibodies
Source: PLoS One. 2014 Nov 11;9(11):e112376. doi: 10.1371/journal.pone.0112376 (PMC4227695; doi:10.1371/journal.pone.0112376)

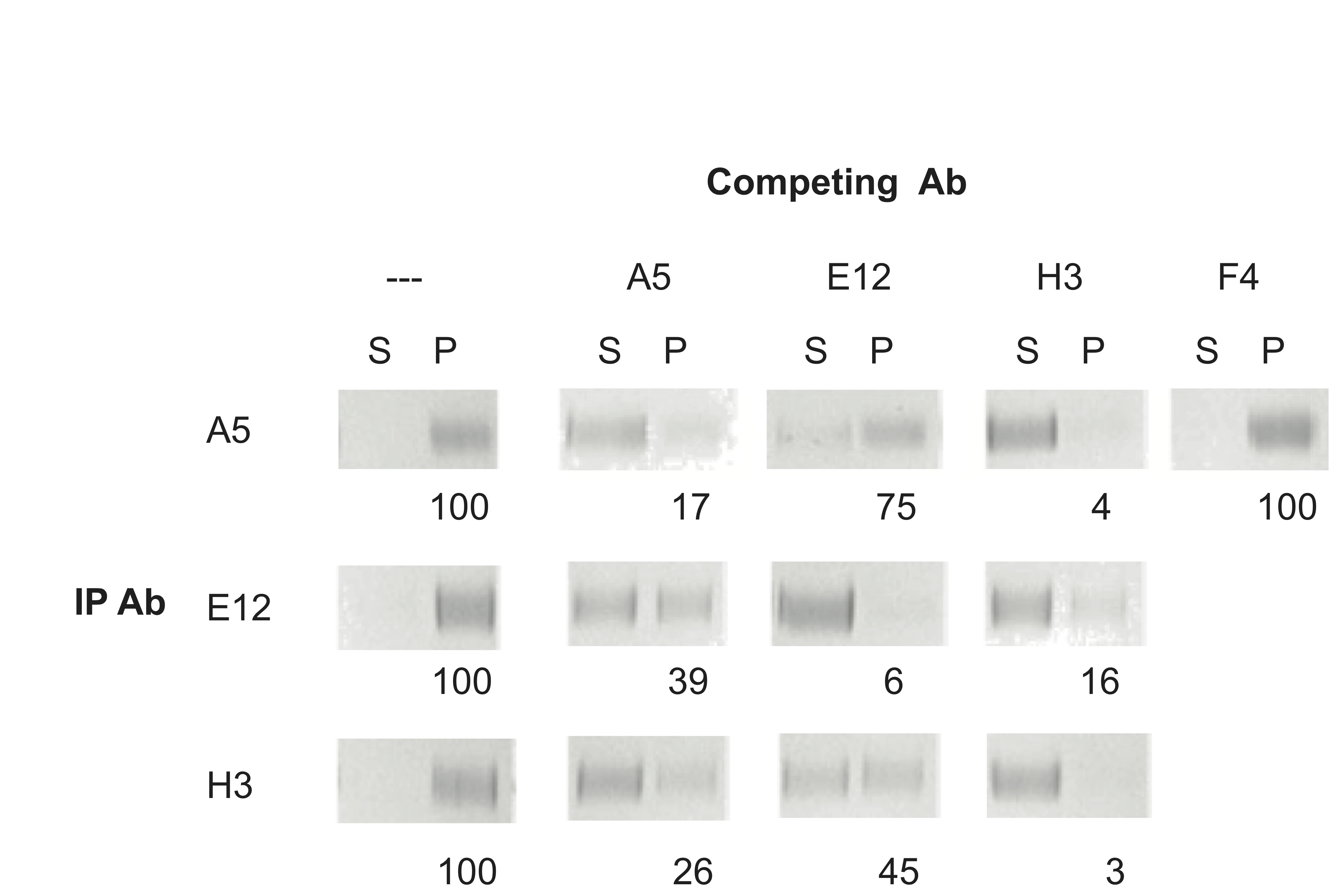

Supplement: Figure S1 — Epitope binning of anti-ERBB3 scFv. A5, E12, and H3 anti-ERBB3 scFv were immobilized on agarose beads and incubated with intact ERBB3 ECD (dI-IV) in the presence of excess free scFv (A5, E12, H3, or F4) to compete for binding as depicted. The impact of competing scFv on immunoprecipitation of dI-IV was evaluated by SDS-PAGE analysis of supernatant and pellet fractions. Numbers obtained by densitometric analysis of the supernatant and pellet fractions represent the percent of dI-IV that was immunoprecipitated by the immobilized ‘IP antibody’ in the presence of excess free ‘competing antibody.’ (TIF) [file pone.0112376.s001.tif]

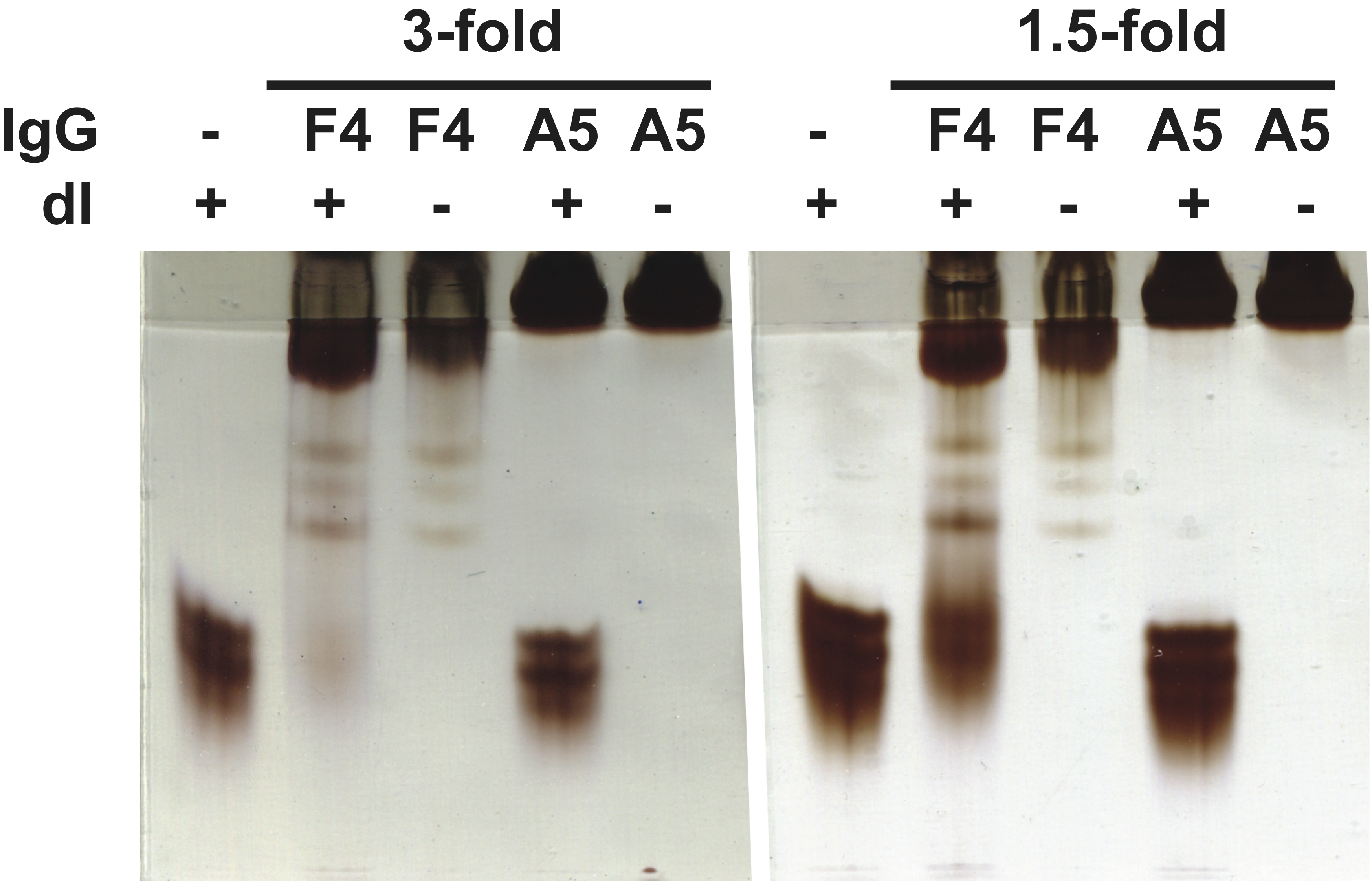

Supplement: Figure S2 — F4 IgG can quantitatively bind dI. F4 and A5 IgGs (30–40 µg) were incubated with dI (2.5–6 µg) at 1.5x and 3x Molar excess of antibody for 30 minutes at room temperature. Binding reactions were resolved on 12.5% native acrylamide gel. As compared to input dI (incubated with equal volume of PBS), incubating with increasing ratio of F4:dI shows dose dependent loss of free dI consistent with quantitative binding. A5 failed to bind under either condition tested. (TIF) [file pone.0112376.s002.tif]

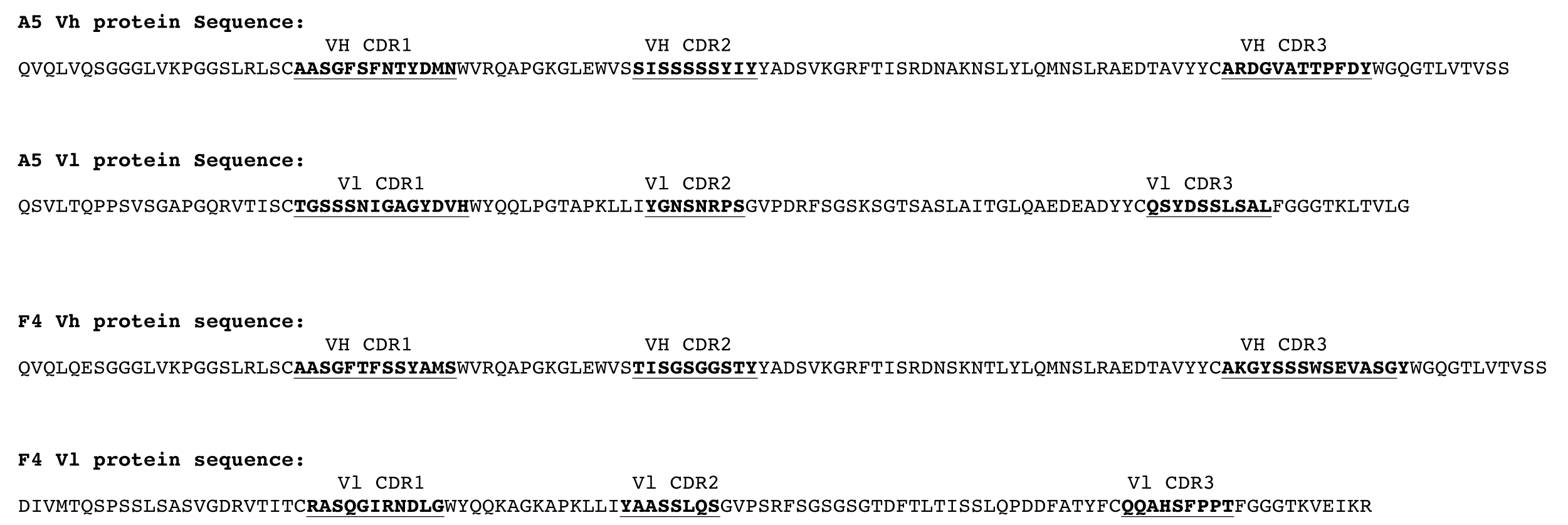

Supplement: Figure S3 — Amino acid sequence of the A5 and F4 variable domains. Amino acid sequences of the variable heavy (Vh) and variable light (Vl) domains for the A5 and F4 IgGs are provided in single letter code. Complementarity Determining Regions (CDRs) are based upon definitions described by North et al [43]. (TIF) [file pone.0112376.s003.tif]

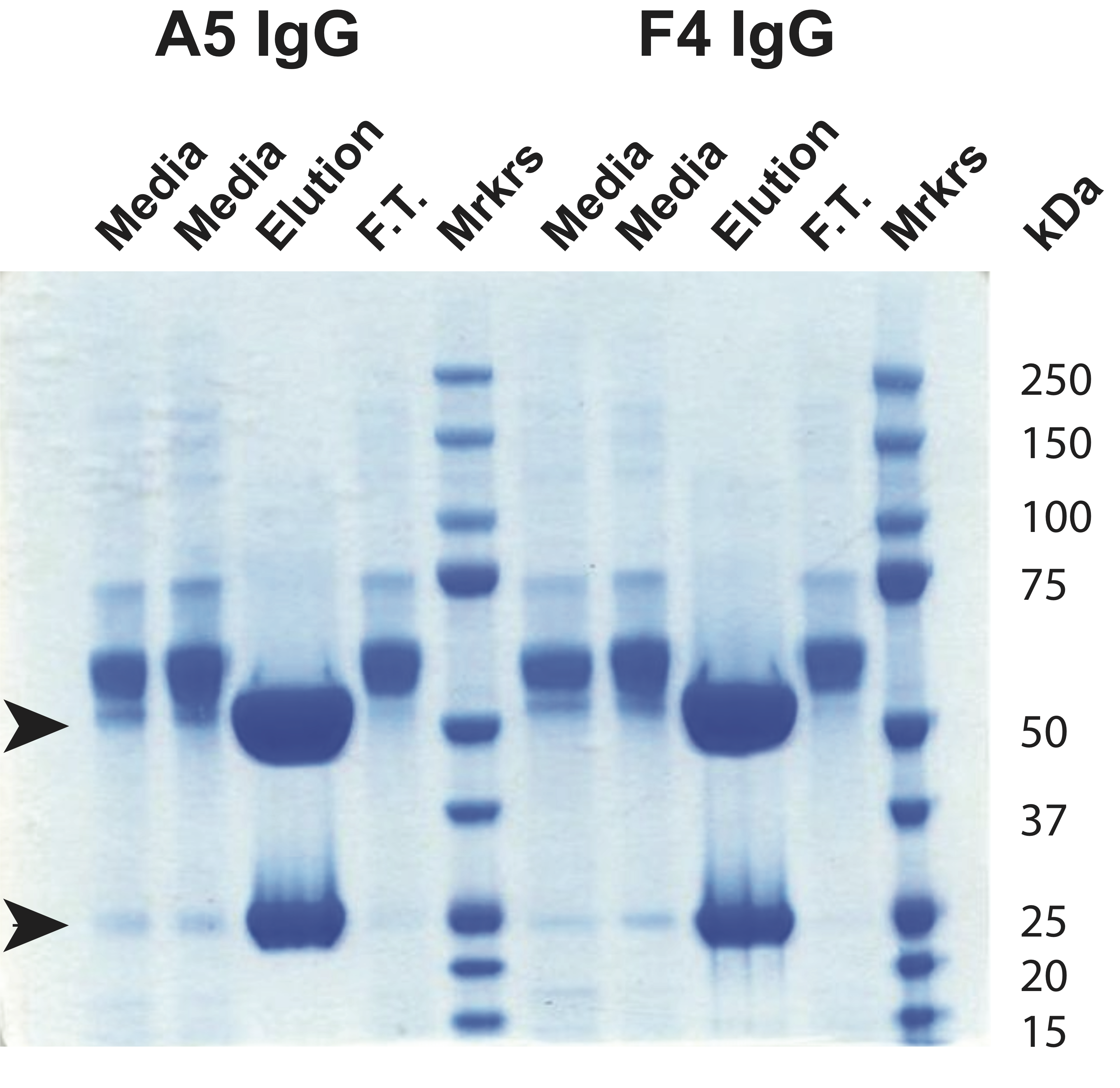

Supplement: Figure S4 — Purification of A5 and F4 IgGs. SDS-PAGE analysis of A5 and F4 IgG purification by protein A chromatography. Arrowheads denote heavy (∼50 kDa) and light (∼25 kDa) chains of expressed IgGs. Media = conditioned media containing expressed IgG, Elution = IgG eluted from protein A column, F.T. = flow through fraction from protein A column, Mrkrs = molecular weight markers. (TIF) [file pone.0112376.s004.tif]

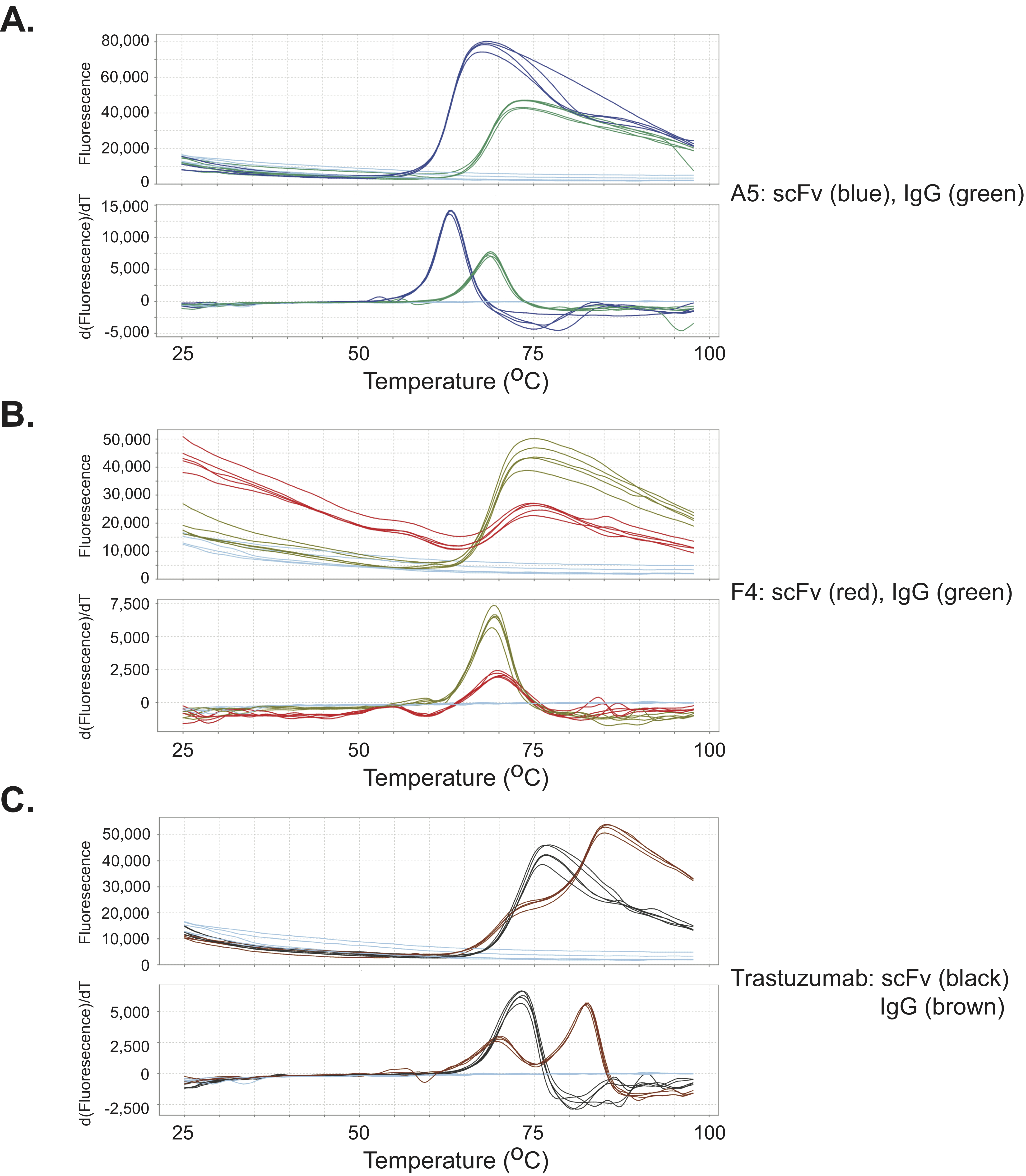

Supplement: Figure S5 — Protein Thermal Shift assay of A5 and F4 IgG. In thermal stability assays the A5 (top panel) and F4 (middle panel) scFv and IgGs were quantified using the Protein Thermal Shift assay (Life Technologies) and manufacturer’s recommended conditions and software. The A5 and F4 IgGs exhibited single transition points at 68.3±0.2°C and 69.2±0.3°C, respectively. This represented a stabilization over the A5 scFv (Tm = 63.3±0.1°C) and was equivalent to the F4 scFv that itself exhibited a Tm of 70±0.2°C. Trastuzumab and an scFv (4D5) engineered based on the trastuzumab amino acid sequence served as controls (bottom panel). Trastuzumab exhibited two major transition points at approximately 70°C and 83°C when analyzed by PTS that are consistent with the 68°C and 80°C melting point transitions obtained by differential scanning calorimetry [44]. Fluorescence and d(Fluorescence)/dT are plotted as a function of temperature. (TIF) [file pone.0112376.s005.tif]

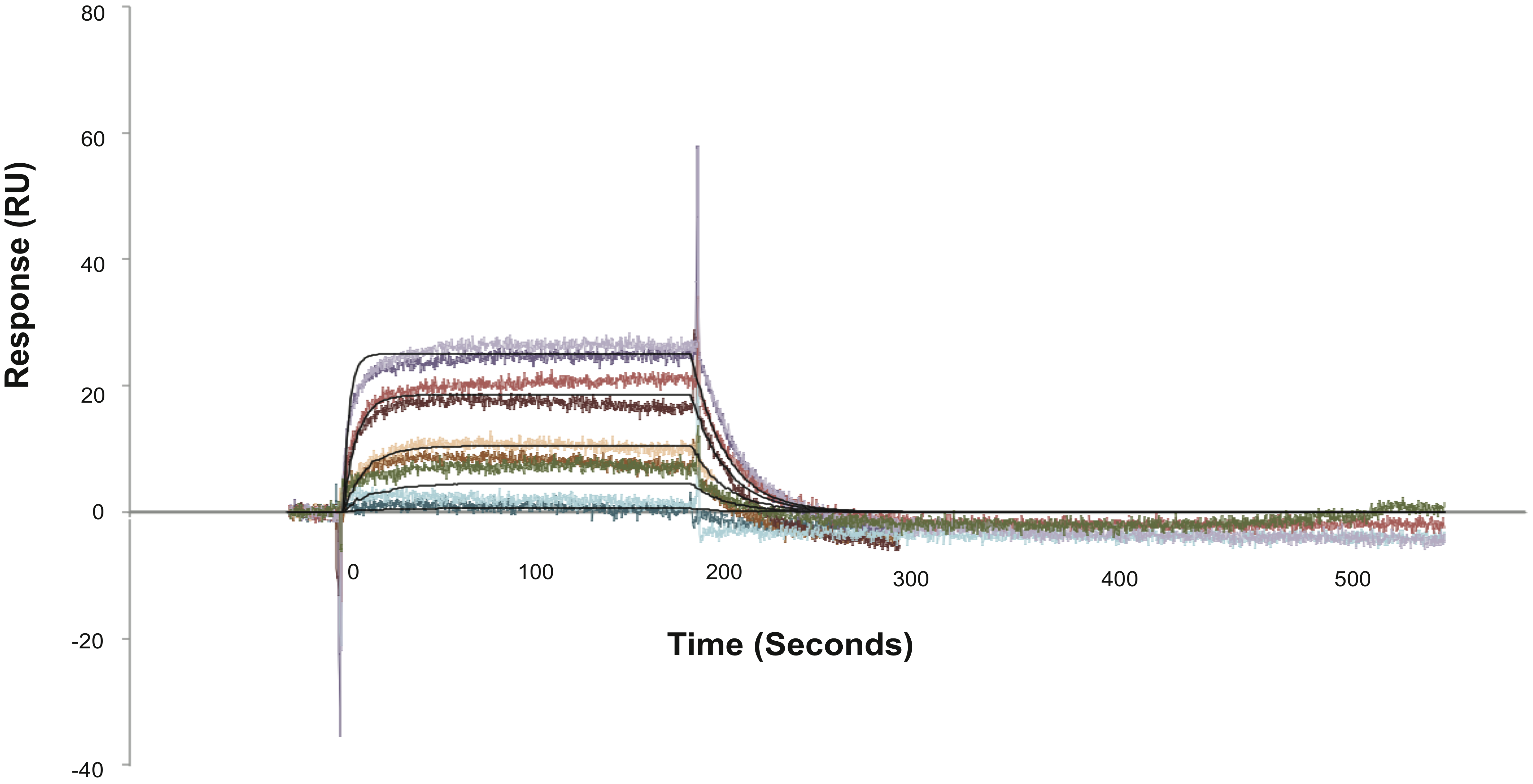

Supplement: Figure S6 — BIAcore analysis of the F4 IgG intrinsic binding affinity. ERBB3 binding by F4 IgG was analyzed by surface Plasmon resonance using a capture-based strategy. F4 IgG (approx 150 RU) was captured on an anti-human Fc surface followed by ERBB3 dI-IV being flowed over the surface. Binding was analyzed at increasing concentrations of ERBB3 dI-IV, in duplicate, and data was fit to a 1∶1 binding model using BIAevaluation. (TIF) [file pone.0112376.s006.tif]

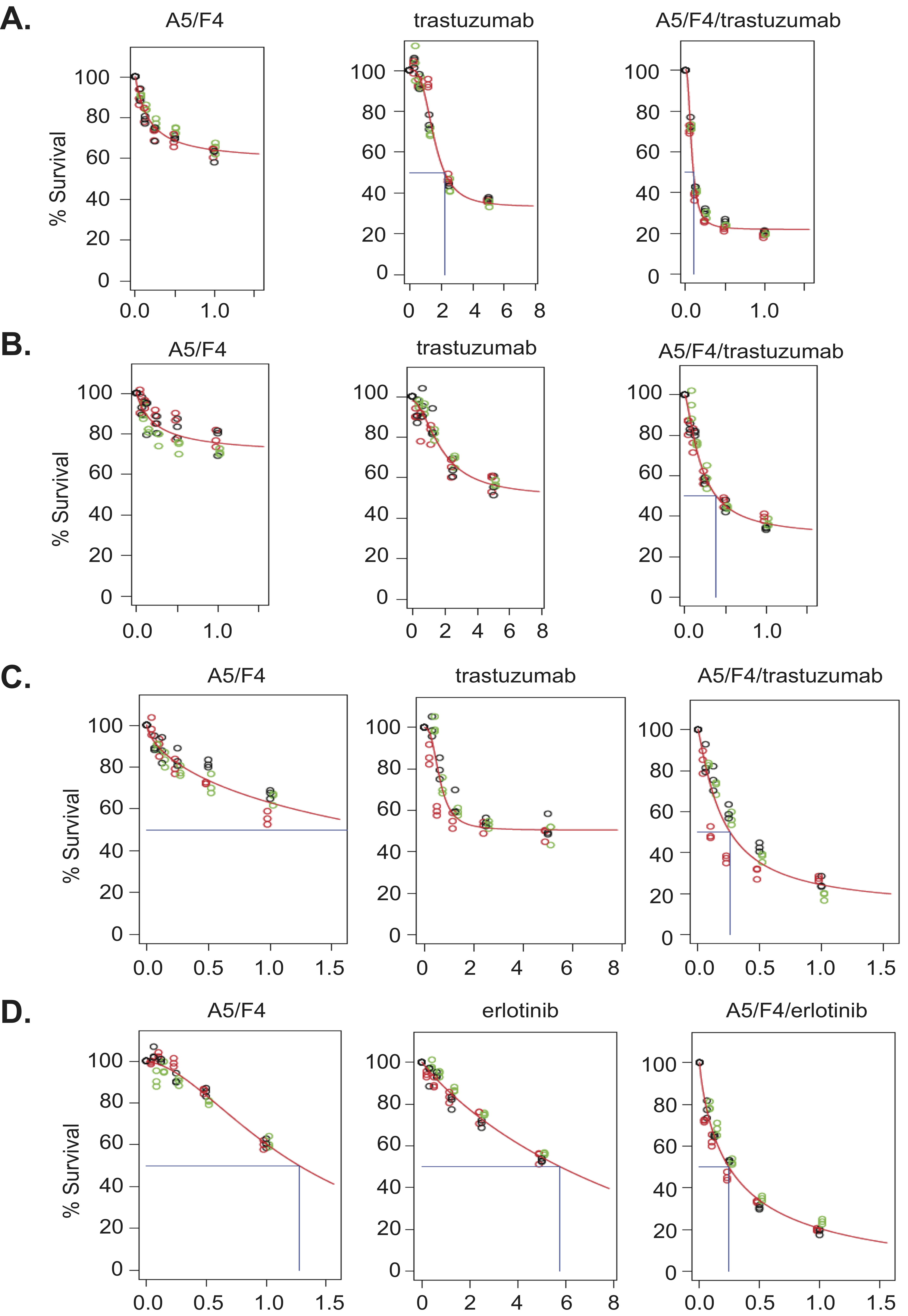

Supplement: Figure S7 — Combination Indices of A5/F4 plus standard of care ERBB-targeted agents. Survival fraction versus dose data for the several drug combinations were fitted using three parameter Hill equations: S(d) = A+(1–A)/[1+(d/d0)∧p], where d is the applied dose and A, d0 and p are parameters. The dose for the fitted equation S(d) = 0.5 was determined for each drug combination and identified as the ID50. Drugs that could not reach 50% kill according to the fitted equation were assigned an infinite ID50 for CI computation. ID50 dose levels, where possible, are indicated in the figure. The combination index was found from CI = d1/D50(1)+d2/D50(2) where LD50(1) or LD50(2) are the estimated ID50s of the single or part-combination of two or the three drugs used. d1 and d2 are the ID50 of the corresponding drugs or part combinations that result in 50% kill using the triple drug combination. In figure 7S–D ACHN cells were inhibited at the 50% level by both the A5/F4 combination alone or by Erlotinib alone. In this case the two ID50s were taken from the first and second plots of D. Synergistic dose, d, was determined from the third plot. In the other three cell lines either the A5/F4 or the Trastuzumab never reach 50% kill. In these one of the two fractions was considered zero by virtue of the assumption that its ID50 was infinite. (TIF) [file pone.0112376.s007.tif]
