# Supplementary material for: Combining Anti-ERBB3 Antibodies Specific for Domain I and Domain III Enhances the Anti-Tumor Activity over the Individual Monoclonal Antibodies
Source: PLoS One. 2014 Nov 11;9(11):e112376. doi: 10.1371/journal.pone.0112376 (PMC4227695; doi:10.1371/journal.pone.0112376)
Supplement: Materials and Methods S1 — (DOC) [file pone.0112376.s008.doc]

**SUPPLEMENTARY MATERIALS AND METHODS**

**Antibody production**

Single-chain Fv antibodies were expressed in TG1 *E. coli* and purified as previously described . The A5 and F4 variable heavy (V_h_) and variable light (V_l_) domains were amplified by polymerase chain reaction (PCR) on BssHII/NheI and BssHII/BsiWI fragments, respectively, and subcloned into the pMAZ-IgH and pMAZ-IgL vectors (gift of Dr. Itai Benhar, Tel Aviv University ). Stable V_l_ hosts were constructed by nucleofection of the pMAZ-IgL constructs into HEK293 cells followed by selection with Hygromycin (0.2mg/mL, Invitrogen cat # 10687-010). A limited pool of V_l_ hosts were then transfected with appropriate pMAZ-IgH constructs, double transfectants were selected in the presence of Hygromycin (0.2mg/mL) and G418 (1mg/mL), and individual clones identified as expressing both the heavy and light chains of their respective IgG were isolated. Individual clones were seeded into HYPERflasks (Corning, cat # 10020) and grown to approximately 90% confluence in pre-production media (DMEM + 10% FBS). Media was then replaced with production media (DMEM + 0.5% FBS) and cells were maintained for one week. Conditioned media, containing secreted IgG, was collected, cell debris pelleted and supernatants passed through a 0.4 μm filter. Clarified media were diluted in Dulbecco’s phosphate buffered saline (PBS, Invitrogen cat # 21600-010) and passed over a HiTrap Protein A HP column (GE Healthcare, cat # 17-5079-02). IgG was eluted with 0.1M citric acid, pH 3 and immediately neutralized with 1 M Tris-HCl. Fractions containing purified IgG, as determined by spectrophotometry and gel electrophoresis, were dialyzed into PBS containing 20% glycerol and stored at -80°C until used. Freezing had no measurable effect on protein activity. IgGs were tested for function by FACS-based cell binding assays. Briefly, BT-474 cells from subconfluent flasks were harvested and resuspended in FACS buffer [PBS, 1% bovine serum albumin, 0.1% sodium azide (w/v)]. Cells (250,000/sample) were incubated with increasing amounts (0, 0.5, 2, and 5 μg) of either A5 or F4 IgG, washed, and bound IgG was detected with goat anti-human IgG-FITC (Jackson ImmunoResearch, cat # 109-095-003). Cells were incubated with 2 μg of PE-conjugated anti-ERBB3 SGP1 antibody (Santa Cruz, cat # sc-53279 PE) as a positive control. Equivalent amounts of mouse IgG1-PE (R&D Systems, cat # IC002P) served as an isotype-matched negative control for SGP1-based detection of ERBB3.

**Thermal Stability of scFv and IgG forms of A5 and F4**

Thermal stability of scFv and IgG were assayed using the protein thermal shift assay (Life Technologies, cat # 4462263) by following the manufacturer’s recommended protocol. Briefly, antibodies were diluted to a final concentration of either 200 μg/mL (A5 scFv, A5 IgG, F4 IgG, 4D5 scFv, and trastuzumab) or 15 μg/mL (F4 scFv) in final volume of 100 μL of dye-containing 1X PTS reaction buffer. Each reaction was aliquoted (20 μL/aliquot) into 5 wells of a MircoAmp Fast Optical 96-well reaction plate (Applied Biosystems, cat # 4346906) and sealed with MicroAmp Optical Adhesive film (Applied Biosystems, cat # 4360954). Reactions lacking either protein or dye served as controls. Samples were run in a 7500 Fast Real-Time PCR system using v2.0.5 software with the reporter set to ROX without a quencher. Thermal profile was determined in a two-step cycle; Step 1: 25ºC for 2 minutes, Step 2: 99ºC for 2 minutes with a standard ramp rate (1%). The derivative Tm for each protein was calculated from the fluorescence melt curves using Protein Thermal Shift Software v1.0.

**Binding affinity**

The intrinsic affinity of the F4 antibody was determined by surface Plasmon resonance using a capture-based approach. Briefly, anti-human Fc polyclonal antibody (SouthernBiotech, catalog # 2040-01) was conjugated to the surface of a CM5 sensor chip (GE Healthcare, cat # BR-1000-12) using standard amine coupling method. Iterative cycles of F4 IgG (5 μL, 85 – 100 RU) followed immediately by increasing concentrations (0 nM – 1000 nM) of ERBB3 ECD (dI – dIV) were flowed over the sensor surface, in duplicate, at a flow rate of 30 μL per minute with a three-minute association phase and a six-minute dissociation phase. The surface was regenerated with two 15-second pulses of 100 mM HCl. An activated and quenched flow cell served as a negative control. Double subtracted data was then fit to a 1:1 Langmuir binding model using BIAevaluation 3.2 to determine kinetic constants.

The apparent affinity of the A5 and F4 IgG were determined in a series of equilibrium binding studies using an ELISA-based approach. Briefly, plates were coated with 5μg/mL concentrations of either ERBB3 or ERBB2 at 4°C overnight and blocked with blocking buffer [1X PBS, 1% powdered milk (w/v)]. Plates were washed and appropriate antibody, diluted in blocking buffer, was incubated at 4°C overnight. Plates were washed, incubated with anti-human FC-horseradish peroxidase (HRP) conjugated antibody (Jackson ImmunoResearch, cat # 109-035-003) for 1 hour at room temp, washed, incubated with substrate, and absorbance read after 30 minutes. Binding to ERBB2 and signal derived from 0 nM concentrations served as background controls and double subtracted data was plotted as absorbance vs concentration.

**REFERENCES**

1. Horak E, Heitner T, Robinson MK, Simmons HH, Garrison J, et al. (2005) Isolation of scFvs to in vitro produced extracellular domains of EGFR family members. Cancer Biother Radiopharm 20: 603-613.

2. Mazor Y, Barnea I, Keydar I, Benhar I (2007) Antibody internalization studied using a novel IgG binding toxin fusion. J Immunol Methods 321: 41-59.
